# Advanced Glycation End Products as a Predictor of Diabetes Mellitus in Chronic Hepatitis C-Related Cirrhosis

**DOI:** 10.3389/fmed.2020.588519

**Published:** 2020-10-26

**Authors:** Ahmed Abdel-Razik, Nasser Mousa, Sahar Zakaria, Mostafa Abdelsalam, Mohamed Eissa, Mohammed I. Abd El-Ghany, Ahmad S. Hasan, Rania Elhelaly, Rasha Elzehery, Niveen El-Wakeel, Waleed Eldars

**Affiliations:** ^1^Tropical Medicine Department, Faculty of Medicine, Mansoura University, Mansoura, Egypt; ^2^Nephrology and Dialysis Unit, Internal Medicine Department, Faculty of Medicine, Mansoura University, Mansoura, Egypt; ^3^Internal Medicine Department, Faculty of Medicine, Mansoura University, Mansoura, Egypt; ^4^Endocrinology and Diabetes Unit, Internal Medicine Department, Faculty of Medicine, Mansoura University, Mansoura, Egypt; ^5^Clinical Pathology Department, Faculty of Medicine, Mansoura University, Mansoura, Egypt; ^6^Medical Microbiology and Immunology Department, Faculty of Medicine, Mansoura University, Mansoura, Egypt

**Keywords:** advanced glycation end products, diabetes mellitus, chronic hepatitis C, liver cirrhosis, insulin resistance

## Abstract

**Background and Aims:** Advanced glycation end products (AGEs) were found to be involved in the pathogenesis of various disorders. Chronic hepatitis C virus infection is the major cause of liver cirrhosis development and glucose metabolism alteration. We aimed to explore the association of AGEs with the development of diabetes mellitus (DM) in patients with cirrhosis in this study.

**Methods:** Only 144 of the 165 non-diabetic patients with cirrhosis were consecutively included in this prospective cohort pilot study, in addition to 72 healthy control subjects. Clinical data and biochemical parameters including basal insulin secretion and insulin sensitivity indices together with AGEs were evaluated in all participants at baseline and every 1 year thereafter for 2 years. Multivariable Cox regression analysis was used to determine the parameters that could predict the development of DM within this period.

**Results:** DM developed in 14 (10%) patients only. Univariate Cox regression analysis showed that AGEs (*P* = 0.004), Homeostatic Model Assessment-Insulin Resistance (HOMA-IR) (*P* = 0.018), HOMA-β (*P* = 0.015), and age (*P* = 0.012) were associated with DM. After adjusting multiple confounders, the multivariable Cox regression model showed that AGEs, HOMA-IR, and age were the strongest variables associated with DM (all *P* < 0.05). Using the receiver operating characteristic curve, AGEs at a cutoff value of more than 82.4 ng/ml had 99.23% specificity, 100% sensitivity, and 0.992 area under the curve (AUC) (all *P* < 0.001) for DM prediction.

**Conclusion:** Our study suggests that AGEs are related to increased incidence of DM, especially in patients with cirrhosis, which is very promising in lowering the risk of DM in these patients.

## Introduction

Chronic hepatitis C virus (HCV) infection is considered the leading cause of liver fibrosis development. Though fibrosis stays asymptomatic for many years, it progresses slowly to cirrhosis and end-stage liver disease and is the major cause of mortality and morbidity related to HCV (1).

Bohan was the first to describe the relationship between diabetes mellitus (DM) and liver cirrhosis (2), and Megyesi named it as hepatogenous diabetes (HD) (3) in which 57% of patients with cirrhosis showed high insulin resistance (IR). Up to 80% of these patients may have glucose intolerance and between 10 and 20% may show diabetes (4).

Advanced glycation end products (AGEs) are defined as a heterogeneous group of irreversibly reactive derivatives. They are formed by non-enzymatic glycation, hence the name, and oxidation of lipids and proteins. They elicit and generate oxidative stress and subsequently induce inflammation (5). They have been tied to the pathogenesis of many diseases (6–11).

It is observed in patients with cirrhosis that the onset of type 2 diabetes is linked to a decrease in insulin secretion rather than an increase though cirrhosis is found to be associated with increased IR leading to increased secretion (12). These associations' interactions have made it difficult to explain the pathogenesis of diabetes in cirrhosis. However, AGE levels independently correlate with IR (13). Our previous clinical data show a close correlation between IR and HCV infection and advanced liver fibrosis (14). These observations led us to believe that AGEs may have a role in DM pathogenesis associated with liver fibrosis/cirrhosis.

To date, this problem has not been discussed properly in the research literature. The objective of this work, therefore, is to assess the possible correlation between AGE and the development of DM in patients with cirrhosis.

## Patients and Methods

This is a cohort prospective pilot study. It was executed in the Department of Tropical Medicine (Mansoura University-Egypt), between October 2015 and August 2019. A total of 306 patients were enrolled consecutively. They had liver cirrhosis and were referred to our department. We included in our study only 165 patients who met the inclusion criteria. All the patients' clinical, hematological, demographic, and biochemical data were assessed and recorded at baseline and over the defined follow-up periods.

The inclusion criteria were patients (1) with liver cirrhosis and (2) 18 years or older. The exclusion criteria were (1) DM and/or HD; (2) impaired fasting glucose (IFG); (3) alcoholic liver disease; (4) liver cirrhosis due to hepatitis B virus, autoimmune hepatitis, NASH, and metabolic or cholestatic liver diseases; (5) collagen vascular diseases; (6) abdominal tuberculosis; (7) uncontrolled thyroid disorders; (8) kidney diseases and hematologic disorders; (9) peritoneal carcinomatosis; (10) pancreatitis; (11) bone marrow suppression; (12) cancers; (13) pregnancy and lactation; (14) osteoporosis; (15) smoking; (16) heart failure; (17) cerebrovascular accidents/Alzheimer's disease; (18) administration of immunosuppressive drugs or drugs likely to affect glucose metabolism; (19) administration of anticoagulant/antiplatelet treatment, hepatotoxic drugs, NSAIDs, and/or oral contraceptive drugs; and finally (20) patients with missing information.

Also, the control group included 72 healthy controls who were sex- and age-matched subjects (male/female = 50/22).

The baseline data were taken during the 1st week of the first visit, while the data for the end of study were taken during the last week of both 1st and 2nd year follow-up periods. The biochemical and radiological findings and clinical examination of our patients at the end of our study did not reveal any complications that would change the parameters in the subjects' health during the follow-up period that were not recorded at the start of the study.

### Diagnosis of Liver Cirrhosis and Its Complications

We diagnosed liver cirrhosis by clinical assessment and biochemical tests, abdominal ultrasonography, liver biopsy for histopathological assessment, elastography, and endoscopic findings implying portal hypertension associated with stigmata of chronic liver disease (15). The cirrhosis severity was given a score according to the Child-Turcotte Pugh (CTP) classification and MELD scoring system (16).

All the cirrhotic complications during the follow-up period were treated based on standardized therapeutic measures (17).

### Treatment

Patients with cirrhosis classified as CTP-A and CTP-B, either naive or treatment-experienced patients, those who did not achieve sustained virologic response (SVR) after treatment with pegylated interferon (Peg IFN) and ribavirin (RBV) or with IFN/Sofosbuvir (SOF), or those who received SOF/RBV only were treated according to EASL guidelines (18).

Our protocol was designed according to the 2015 EASL recommendations for hepatitis C treatment (18), which approved that HCV genotype 4 infected patients should be treated with SOF (400 mg) and daclatasvir (DCV) (60 mg) once daily for 12 weeks, besides adding RBV daily based on their weight, i.e., 1,000 mg for patients <75 kg or 1,200 mg for those ≥75 kg. We adjusted the RBV dose according to the estimated glomerular filtration rate (eGFR). We also extended the treatment of the patients who were contra-indicated to administer RBV to 24 weeks (18).

Treatment-experienced patients can be re-treated with a SOF combined with DCV for 12 weeks with RBV or 24 weeks without it (18).

### Monitoring of Treatment Efficacy

Hepatitis C viral RNA level was measured at baseline, through screening, at the end of treatment (either 12 or 24 weeks), and finally 12 weeks after that. We used Roche COBAS TaqMan HCV assay version 2.0 to measure the HCV RNA, which has a detection limit of 15 IU/ml. The primary virological outcome was to achieve SVR12, where the virus is undetected (below detection limit) for 12 or more weeks. On the contrary, virological failure is categorized as either non-response (HCV RNA is still detectable at the end of the treatment period) or relapse (HCV RNA becomes detectable again during follow-up after being undetectable at the end of the treatment period) (19).

AGE levels were measured at baseline and again after the completion of treatment by 12 weeks.

### Safety Assessments

Safety was evaluated through laboratory tests, physical examinations, and reports of clinical adverse events at scheduled clinic visits and then treated according to the study schedule.

### Diagnosis of DM

#### Diabetes

Patients were diagnosed with diabetes through clinical examination, history of antidiabetics administration, and/or fasting plasma glucose (FPG) of 126 mg/dl or more (≥7 mmol/l) (20).

Patients with HD usually show normal FPG, but abnormal response to an oral glucose tolerance test (OGTT), when at least two of the following three plasma glucose levels (measured during OGTT) are met or exceeded: fasting: 95 mg/dl (5.3 mmol/L), 1 h: 180 mg/dl (10.0 mmol/L), and 2 h: 155 mg/dl (8.6 mmol/L), which is mandatory for the diagnosis (3).

Hypersplenism in patients with liver cirrhosis has played a role in shortening erythrocyte life span and falsely lowering levels of HbA1c. Therefore, OGTT is required to identify DM or IGT in cirrhosis. Subjects with normal HbA1c (and FPG) and abnormal OGTT are likely to be those with HD; however, in most patients with increased FPG levels, diabetes is commonly type 2 DM.

#### IFG or Prediabetes

Patients with fasting blood sugar of 110 mg/dl or more (≥6.1 mmol/L) but <126 mg/dl (<7 mmol/L) were considered prediabetic with IFG and were advised to have a glucose tolerance blood test (21).

We followed the García-Compeán et al. (22) recommendation in treating all patients who developed DM.

### Data Collection

All our study data were collected by trained investigators. All patients were asked to complete a self-validated, standardized questionnaire that allowed us to collect information on their smoking habits, alcohol consumption, and medical and therapeutic history, especially malignancy, hypertension, and diabetes history if present. We used the formula $\frac{weight\;\left(kg\right)}{{height\;\left(m\right)}^{2}}$ to calculate BMI.

In our study, patients were considered vegetarian if they refrain from eating meat, but have two to three vegetable servings or five fruits servings during the day. One vegetable serving is equivalent to a cup of raw green leafy vegetables or ½ cup of other cooked or chopped raw vegetables while one fruit serving is equivalent to a medium-sized banana, orange, or apple, or ½ cup of canned/chopped fruit or juice (23).

We used the World Health Organization-developed Global Physical Activity Questionnaire (GPAQ), used in the STEPS questionnaire, to assess the physical activity. This questionnaire divides the physical activity into three distinct intensity levels: light, moderate, and vigorous according to three different behavioral actions: transport, work, and during leisure time (23).

Participants were considered sufficiently active when fulfilling or exceeding the minimum duration and intensity of physical activity every week per WHO recommendations, which are moderate-intensity for 150 min, vigorous-intensity for 75 min of or an equivalent combination of both to reach at least 600 MET min per week with each activity performed in at least 10-min long sessions (24).

### Sampling

Six milliliters of fresh venous blood was aspirated from all participants following overnight fasting and divided into 4 ml with separating gel for serum samples and 2 ml on EDTA for full blood count (FBC). The serum samples were centrifuged for 10 min at 1,000–3,000 RPM, and then the serum was collected and divided into aliquots and kept in a −20°C freezer until later analysis.

### Methodology

We used the Dimension Xpand Plus chemistry analyzer (Siemens Technology, Princeton, New Jersey) to assess serum creatinine and complete liver function tests, the CELL-DYN Emerald 22 Hematology Analyzer (Abbott, Wiesbaden, Germany) to assess complete blood count (CBC), the Spinreact kits [Sant Esteve De Bas (GI), Spain] to measure serum triglycerides (TG) and cholesterol, and enzyme-linked immunosorbent assay (ELISA) kits to measure the fasting serum insulin (FSI) levels (Calbiotech, Spring Valley, California), plasma AGEs (MyBioSource, San Diego, CA 92195-3308, USA; Cat No. MBS267540), and serum C-peptide [DiaMetra, Garibaldi, 18 20090 SEGRATE (MI) Italy; Cat No. DKO077]. As for measuring the hemoglobin A1c (HbA1c), we used ion exchange resin chromatography kits (StanBio, 1261 North Main street, Boerne, Texas USA) procedure No (0350). The eGFR is calculated by the abbreviated Modification of Diet in Renal Disease (MDRD) study equation: $186\times Age^{-0.203}\times {\left(\frac{Creatinine}{88.4}\right)}^{-1.154}\;\times \left(0.742\;if\;female\right)\times \left(1.210\;if\;black\right)$ (25).

All participants underwent an OGTT at baseline and at the end of the 1st and 2nd year follow-up periods. Patients developing diabetes were confirmed with OGTT for proper diagnosis during the follow-up period.

We used the following HOMA formulas to calculate the basal insulin secretion and sensitivity indices (26):

β-cell function (HOMA-β) = $\frac{FSI\times 360}{FPG-63}$

Insulin resistance (HOMA-IR) = $\frac{FSI\times FPG}{405}$

### Detection of HCV Genotype IV

Hepatitis C viral RNA was extracted from patients' sera using QIAamp Viral RNA Mini Kit (Qiagen, Hilden, Germany). First, we amplified the core region by RT-PCR in the Biometra thermal cycler (Analytik Jena Company, Germany) using specific primers common to all genotypes (27); then, we ran a second PCR using primers specific to HCV-4 (27) and Taq DNA polymerase (Qiagen, Hilden, Germany). HCV-4 was determined by measuring the amplified product against a Thermo Fisher 100-bp DNA ladder marker (Life Technologies, USA). Genotype IV-specific band was detected at position 99 bp.

### Ethics

All procedures and the study protocols were evaluated and accepted by the Mansoura Institutional Review Board. In addition, informed consents were obtained from all participants. The study was carried out in agreement with the Helsinki Declaration's recommendations.

### Statistical Analysis

Our results were plotted and analyzed by the Social Package of Statistical Science (SPSS) software version 20 (SPSS Inc., Chicago, IL, USA). Quantitative data are described as mean ± SD. We used the Kolmogorov–Smirnov test to determine the compatibility of normally distributed data. We also used Mann–Whitney *U* test, Student *t*-test, and χ2 test to analyze non-normally distributed, normally distributed, and categorical data, respectively. Spearman's correlation analysis was carried out between AGE levels and other variables. Variables with a *P* < 0.05 in the univariate analysis were enrolled in the multivariable Cox regression analysis. Univariate and multivariable Cox regression models were assessed to identify the independent variables that could be utilized to predict the development of DM. The receiver operating characteristic (ROC) curve and area under the curve (AUC) were performed, and the best cutoff values were calculated to predict the development of DM. A two-tailed *P* < 0.05 was considered significant.

## Results

### Patient Characteristics

**Figure 1:**
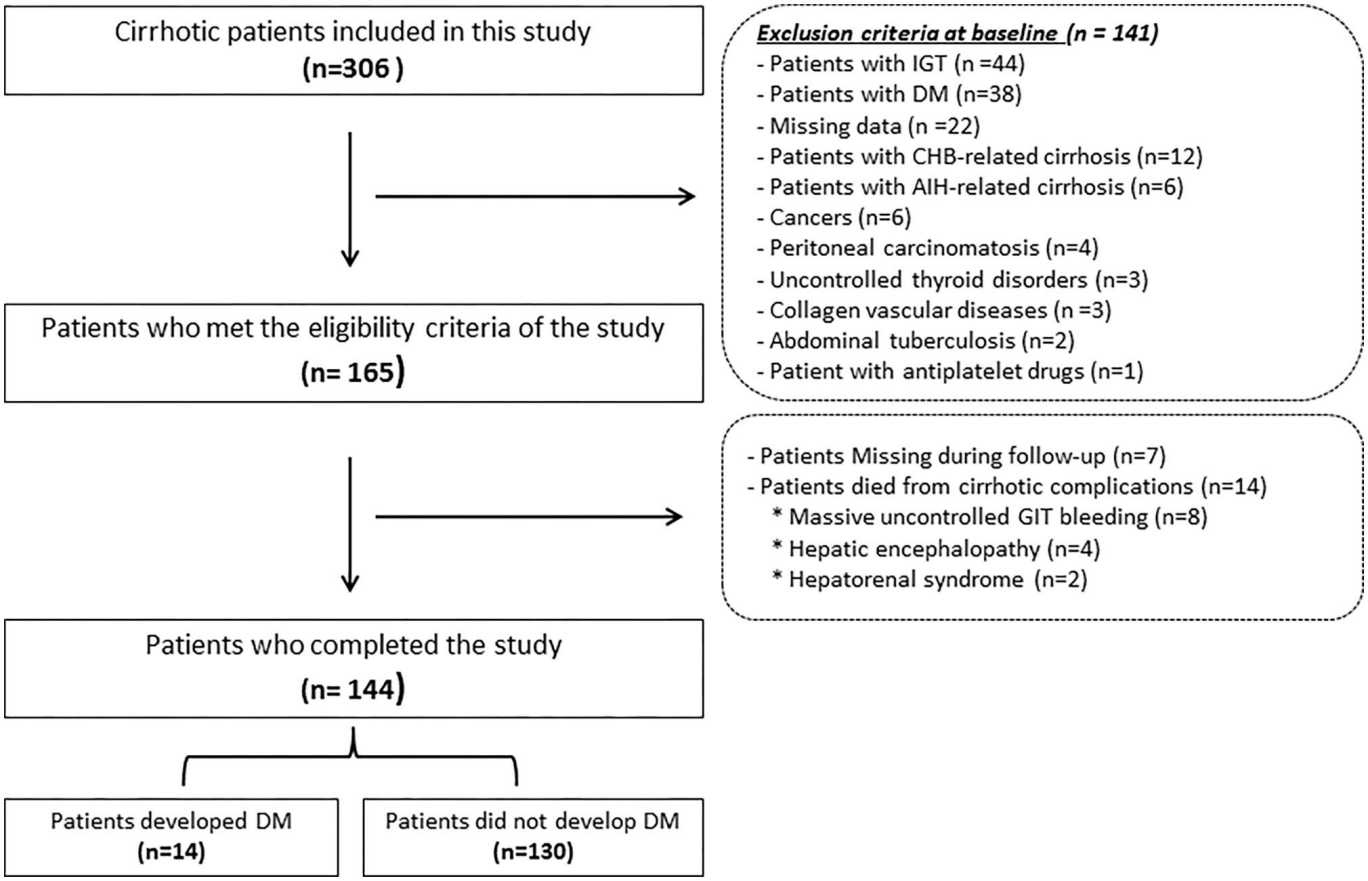
Flowchart of the patients included in this study. IGT, Impaired glucose tolerance; DM, diabetes mellitus; CHB, chronic hepatitis B; AIH, autoimmune hepatitis; GIT, Gastrointestinal tract.

Only 144 patients out of the enrolled 165 who fulfilled the inclusion criteria have completed the study where 7 were dropped due to non-compliance along the follow-up period and 14 died from complications of cirrhosis, e.g., hepatorenal syndrome (*n* = 2), massive uncontrolled GIT bleeding (*n* = 8), and hepatic encephalopathy (*n* = 4) as shown in Figure 1. Table 1 lists the biochemical, clinical, and demographic characteristics of the participants enrolled at the beginning of this study. Patients showed a statistically significant increase in AST, ALT, GGT, ALP, serum bilirubin, INR, serum creatinine, C-peptide, HOMA-IR, and AGEs compared to that of the control group (all *P* < 0.05). However, they showed a statistically significant decrease in serum albumin, hemoglobin, platelet count, and white cell count, compared to that of the control group (*P* < 0.001).

**Table 1:** Baseline clinical, demographic, and biochemical characteristics of enrolled participants.

**Characteristics**	**Patients with cirrhosis (*n* = 144)**	**Control group (*n* = 72)**	***P*-value**
Age (years)	57.6 ± 5.5	56.2 ± 4.9	0.07
Sex (male/female)	93/51	50/22	0.82
Diet			
Vegetarian	58 (40.3)	32 (44.4)	0.57
Non-vegetarian	86 (59.7)	40 (55.6)	0.56
Body mass index (kg/m^2^)	26 ± 1.1	25.8 ± 0.4	0.14
Physical activity			
≥600 MET min/week	32 (22.2)	18 (25)	0.64
<600 MET min/week	112 (77.8)	54 (75)	0.65
Hypertension			
Present	6 (4.2)	–	–
Absent	138 (95.8)	72 (100)	0.08
Family history of diabetes			
Absent	130 (90.3)	66 (91.7)	0.74
Present	14 (9.7)	6 (8.3)	0.73
Patients receiving DAA (*n* = 114)			
SVR	90 (79)	–	–
Non-responder	24 (21)	–	–
Patients not receiving DAA	30	–	–
Hemoglobin (gm/dl)	9.2 ± 0.7	12 ± 0.4	<0.001
WBCs (×10^3^/cm^2^)	3.5 ± 0.4	6.2 ± 1.6	<0.001
Platelet count (×10^3^/cm^2^)	60.6 ± 24.8	196.6 ± 47.5	<0.001
Total cholesterol (mg/dl)	178.1 ± 13.9	175.4 ± 10.2	0.15
Triglyceride (mg/dl)	122.8 ± 16	125.8 ± 9.1	0.14
AST (U/L)	42.2 ± 14.6	28 ± 5.4	<0.001
ALT (U/L)	39.4 ± 13.9	28.3 ± 4.7	<0.001
GGT (U/L)	38.5 ± 11	21.6 ± 5.1	<0.001
ALP (IU/ml)	102.4 ± 15.2	53.8 ± 11.3	<0.001
Bilirubin (mg/dl)	2.5 ± 1.3	0.9 ± 0.1	<0.001
Albumin (g/dl)	3 ± 0.4	4.2 ± 0.2	<0.001
INR	1.5 ± 0.3	0.9 ± 0.1	<0.001
Creatinine (mg/dl)	1.2 ± 0.3	0.9 ± 0.2	<0.001
eGFR	77.3 ± 9.5	95.7 ± 6.1	<0.001
Child-Pugh score	7.7 ± 2.6	–	–
MELD score	16.8 ± 4	–	–
FPG (mg/dl)	88.3 ± 7.7	87.3 ± 4.8	0.31
HbA1c	4.52 ± 0.56	4.41 ± 0.56	0.18
C-peptide (ng/ml)	4.28 ± 0.68	2.8 ± 0.32	<0.001
Insulin (mIU/ml)	16.1 ± 2.3	8.7 ± 1.5	<0.001
HOMA-IR	3.8 ± 0.5	1.9 ± 0.2	<0.001
HOMA-β	135.7 ± 11.6	138.4 ± 8.4	0.08
AGEs (ng/ml)	77.8 ± 54.3	14.3 ± 1.5	<0.001

Data were presented as mean ± SD or n (%).

*DAA, direct acting antiviral; ALP, alkaline phosphatase; HOMA-β, homeostasis model assessment of β-cell function; SVR, sustained virological response; INR, international normalized ratio; AGEs, advanced glycation end products; WBCs, white blood cells; eGFR, estimated glomerular filtration rate; AST, aspartate aminotransferase; MELD, Model for End-Stage Liver Disease; ALT, alanine aminotransferase; FPG, fasting plasma glucose; GGT, γ-glutamyl transpeptidase; HbA1c, glycated hemoglobin; HOMA-IR, homeostasis model assessment-insulin resistance*.

We followed up the participants by hematological and biochemical blood tests every year for 2 years after the initial baseline assessment.

**Figure 2:**
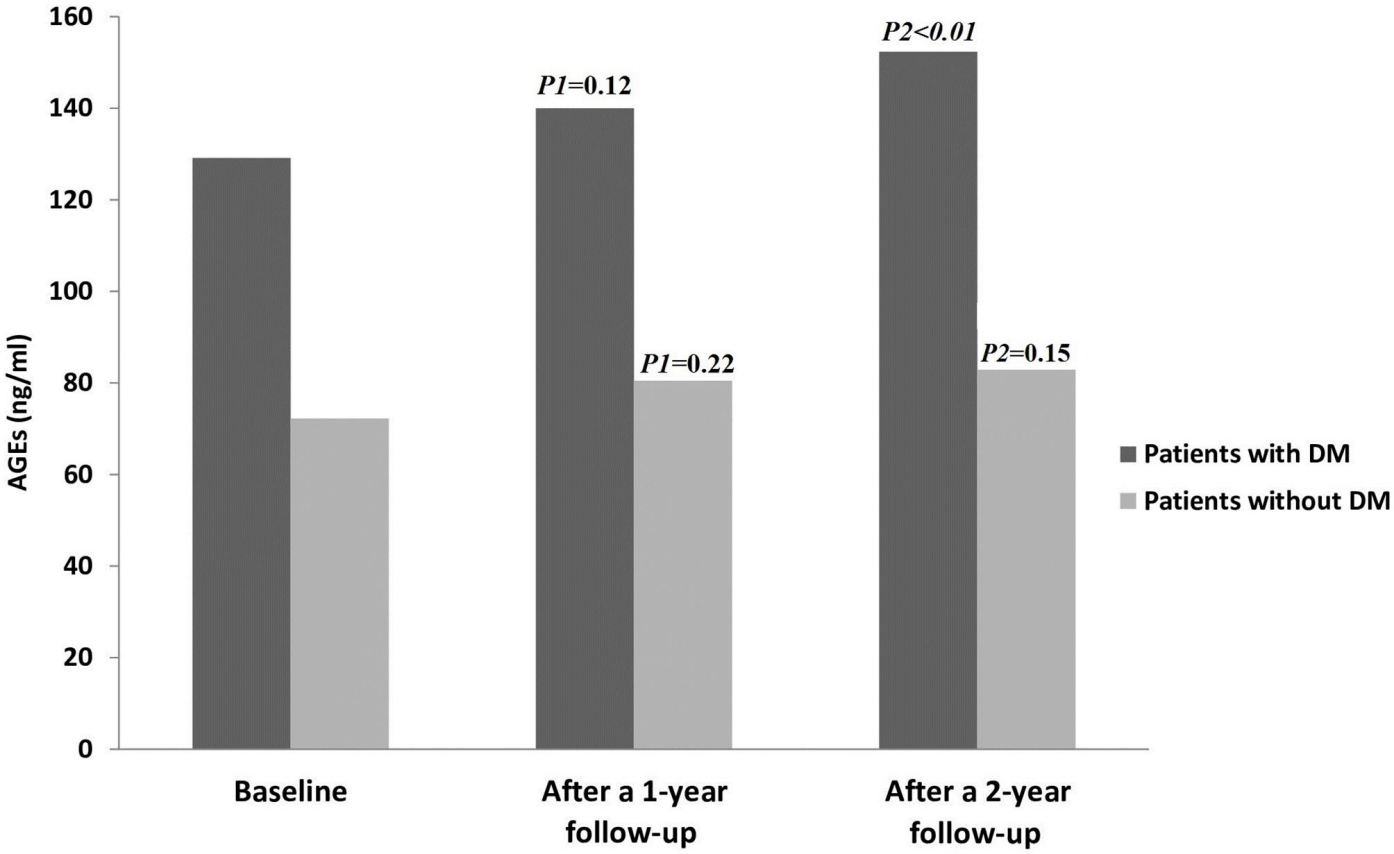
AGE levels in all patients throughout the study period. AGEs, Advanced glycation end products; DM, diabetes mellitus. *P1* = Baseline vs. after a 1-year follow-up; *P2*= Baseline vs. after a 2-year follow-up.

Fourteen patients (10%; CTP-A = 3, CTP-B = 6, and CTP-C = 5) out of all the patients examined during the follow-up period (*n* = 144) developed DM, but none in the control group. Figure 2 displays AGE levels in patients with and without DM.

In the control group, there were no statistically significant changes in AGE levels between the baseline and 1 year follow up, as well as at the end of study (15.3 ± 2.2 vs. 15.8 ± 2.25; *P* = 0.18 and 15.3 ± 2.2 vs. 16 ± 2.2; *P* = 0.06), respectively. Also, there were no statistically significant changes in all parameters between the baseline and at the end of the study (all *P* > 0.05) (data not shown).

### Correlation of AGEs With Clinical and Biochemical Patterns of the Studied Patients

Spearman correlation analysis showed that there were significant positive correlations between AGEs and age (*rho* = 0.63, *P* = 0.001), CTP (*rho* = 0.59, *P* = 0.011), MELD scores (*rho* = 0.71, *P* < 0.001), and HOMA-IR (*rho* = 0.68, *P* < 0.001), while there was a significant inverse correlation between AGEs and HOMA-β (*rho* = –0.57, *P* = 0.012).

### Univariate and Multivariable Cox Regression Models Predicting DM Within 2 Years Follow-Up

Table 2 lists the demographic, biochemical, and clinical parameters of patients with and without DM.

**Table 2:** Clinical, demographic, and biochemical characteristics of patients with and without DM during the follow-up period.

**Characteristics**	**Patients with DM (*n* = 14)**	**Patients without DM (*n* = 130)**	***P*-value**
Age (years)	55.8 ± 5.3	58.4 ± 5.5	0.09
Sex (male/female)	9/5	84/46	0.99
Diet			
Vegetarian	6 (42.9)	52(40)	0.83
Non-vegetarian	8 (57.1)	78 (60)	0.84
Body mass index (kg/m^2^)	26.6 ± 1.2	26 ± 1.1	0.07
Physical activity			
≥600 MET min/week	3 (21.4)	29 (22.3)	0.94
<600 MET min/week	11 (78.6)	101 (77.7)	0.93
Hypertension			
Present	1 (7.1)	5 (3.8)	0.55
Absent	13 (92.9)	125 (96.2)	0.6
Family history of diabetes			
Absent	12 (86)	118 (91)	0.55
Present	2 (14)	12 (9)	0.56
Patients receiving DAA			
SVR	9 (64.3)	81 (62.3)	0.88
Non-responder	2 (14.3)	22 (16.9)	0.8
Patients not receiving DAA	3 (21.4)	27 (20.8)	0.96
Hemoglobin (gm/dl)	9.1 ± 0.7	9.2 ± 0.7	0.61
WBCs (×10^3^/cm^2^)	3.5 ± 0.3	3.5 ± 0.4	0.99
Platelet count (×10^3^/cm^2^)	87 ± 36	57.5 ± 21.8	<0.001
Total cholesterol (mg/dl)	180.4 ± 10.9	177.8 ± 14.2	0.51
Triglyceride (mg/dl)	128.6 ± 11.2	122.1 ± 16.3	0.15
AST (U/L)	48.3 ± 21.6	41.6 ± 13.6	0.1
ALT (U/L)	45.9 ± 21.4	38.7 ± 12.8	0.07
GGT (U/L)	36.3 ± 7.6	38.8 ± 11.2	0.42
ALP (IU/ml)	112.8 ± 17.4	101.3 ± 14.5	0.007
Bilirubin (mg/dl)	2.7 ± 1.6	2.5 ± 1.3	0.6
Albumin (g/dl)	3.4 ± 0.5	3 ± 0.4	<0.001
INR	1.5 ± 0.3	1.6 ± 0.3	0.23
Creatinine (mg/dl)	1.2 ± 0.3	1.3 ± 0.3	0.24
eGFR	76.6 ± 9.3	79.4 ± 10	0.32
Child-Pugh score	7.6 ± 2.4	7.7 ± 2.6	0.89
MELD score	16.4 ± 4.8	16.8 ± 3.8	0.72
FPG (mg/dl)	95.6 ± 3.9	87.5 ± 7.6	<0.001
HbA1c	4.68 ± 0.58	4.45 ± 0.53	0.16
C-peptide (ng/ml)	3.95 ± 0.88	3.29 ± 1.9	0.2
Insulin (mIU/ml)	17.7 ± 1.9	16.9 ± 1.7	0.1
HOMA-IR	4.2 ± 0.4	3.8 ± 0.5	0.004
HOMA-β	140 ± 12.3	135.3 ± 11.5	0.15
AGE (ng/ml)	129.1 ± 17.8	72.2 ± 54.1	<0.001

Data were presented as mean ± SD or n (%).

*DAA, direct acting antiviral; ALP, alkaline phosphatase; HOMA-β, homeostasis model assessment of β-cell function; SVR, sustained virological response; INR, international normalized ratio; AGEs, advanced glycation end products; WBCs, white blood cells; eGFR, estimated glomerular filtration rate; AST, aspartate aminotransferase; MELD, Model for End-Stage Liver Disease; ALT, alanine aminotransferase; FPG, fasting plasma glucose; GGT, γ-glutamyl transpeptidase; HbA1c, glycated hemoglobin; HOMA-IR, homeostasis model assessment-insulin resistance*.

There is no significant difference in sex, age, diet, BMI, physical activity, family history of diabetes, hypertension, patients receiving DAA, hemoglobin, WBCs, total cholesterol, serum TG, AST, ALT, GGT, serum bilirubin, INR, serum creatinine, eGFR, serum TG, HbA1c, C-peptide, serum insulin, CTP and MELD classifications, or HOMA-β between the two groups (all *P* > 0.05). Univariate Cox regression analysis revealed a statistically significant difference in AGEs, HOMA-IR, HOMA-β, and age in both our groups (all *P* < 0.05) in Table 3.

**Table 3:** Univariate and multivariable Cox regression analysis models in the studied patients to predict DM development.

	**Univariate Cox regression**			**Multivariable Cox regression**		
	**HR**	**95% CI**	***P*-value**	**HR**	**95% CI**	***P*-value**
AGE	1.004	1.0014 to 1.0073	0.004	1.005	1.0014 to 1.0083	0.006
HOMA-IR	3.412	1.2437 to 9.3617	0.018	3.355	1.1635 to 9.6719	0.026
HOMA-β	1.036	0.9872 to 1.0874	0.015	–	–	–
Age	0.9301	0.8481 to 1.0202	0.012	0.901	0.8177 to 0.9916	0.034

*AGEs, advanced glycation end products; HOMA-IR, homeostasis model assessment-insulin resistance; HOMA-β, homeostasis model assessment of β-cell function; DM, diabetes mellitus; CI, confidence interval; HR, hazard ratio*.

We re-evaluated the multivariable Cox regression analysis model after adjusting multiple confounders using the formerly described parameters at baseline related to the development of DM during the 2 year follow-up period. This revealed that the only factors independently associated with DM development are AGEs, HOMA-IR, and age (Table 3).

**Figure 3:**
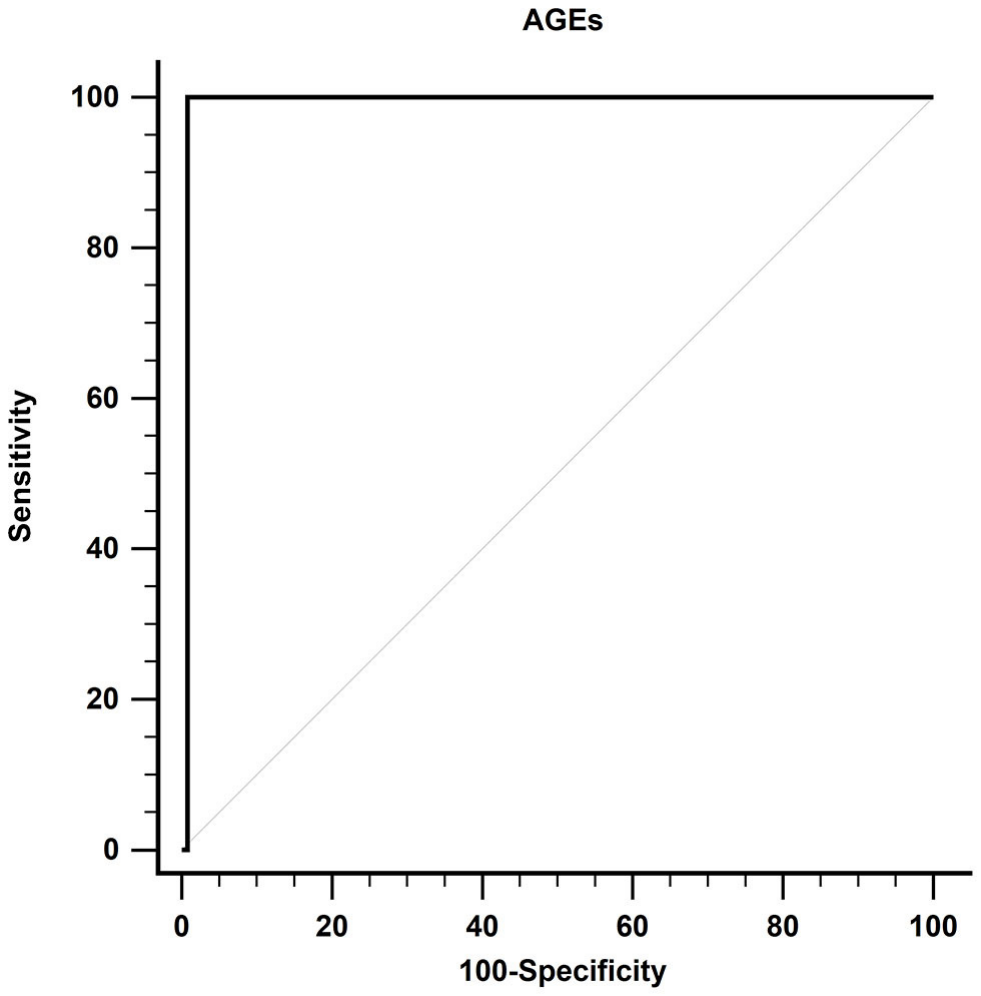
Receiver operating characteristic curve of AGEs in detecting DM in patients with cirrhosis. AGEs, advanced glycation end products; DM, diabetes mellitus.

Using the ROC curve, at a cutoff value of more than 82.4 ng/ml, AGEs had 99.23% specificity, 100% sensitivity, 0.992 AUC, 93.33% positive predictive value (PPV), 100% negative predictive value (NPV), and *P* < 0.001 for prediction of DM, as shown in Figure 3.

### Therapeutic Findings of DAAs and Antidiabetic Agents

Patients with CTP-A (*n* = 62) and CTP-B (*n* = 52) cirrhosis received therapy according to our study protocol. SVR was reported in 90 (79%) patients. There was no significant difference between AGEs at baseline and 12 weeks after completion of treatment (73.8 ± 20.8 vs. 78.6 ± 21.4; *P* = 0.1).

There were no significant differences in AGEs at baseline and end of therapy regarding SVR (*n* = 90) (75.6 ± 21.3 vs. 80.2 ± 21.6; *P* = 0.15), non-responder (*n* = 15) (78.6 ± 21.5 vs. 82.1 ± 21.8; *P*= 0.66), or relapse (*n* = 9) (76.5 ± 20.2 vs. 80.6 ± 21.2; *P* = 0.68).

Regarding the development of DM, there was no statistically significant difference between patients who received therapy and those who did not (11/114 *vs*. 3/30; *P* = 0.95), respectively.

Two patients (CPT-A) were treated with oral hypoglycemic agents (Glimepiride 1 mg) while 12 patients (CPT-B and CPT-C) were treated with insulin.

### Safety and Tolerability of DAAs and Antidiabetic Agents

The most common adverse events were fatigue (8 and 28.8%), anemia (14.5 and 26.9%), headache (6.5 and 15.4%), RBV dose reductions (4.8 and 9.6%), hyperbilirubinemia (11.3 and 23.1%), and pruritus (6.5 and 11.5%) in CTP-A and CTP-B patients, respectively.

We saw more serious side effects in CTP-B patients than in CTP-A; 6.5 and 9.6% developed ascites, 1.6 and 5.8% had hepatic encephalopathy, 3.2 and 7.7% had GIT bleeding, 0.0 and 3.8% had renal impairment, and 6.5 and 9.6% had hepatocellular carcinoma (HCC) in CTP-A and CTP-B, respectively.

No adverse effects related to the treatment of DM were reported during the follow-up period regarding the drugs' safety and tolerability.

## Discussion

AGEs were recently proven to play a role in the fibrosis and/or cirrhosis of CHC patients through autophagy induction and hepatic stellate cells (HSC) activation (28, 29). This process generates irreversible glycation end products, which in turn induce cellular anomalies that end in more fatal clinical consequences. This glycation reaction also activates multiple cellular signals through different receptors, and their toxic by-products result in acceleration of the pathogenesis of many disorders (30–32).

The liver is the main site for AGE catabolism and clearance from the circulation; in one study, it successfully removed more than 90% of AGEs intravenously injected in rats through endocytosis (33, 34), and thus the AGE–Receptor for Advanced Glycation Endproducts (RAGE) axis plays an important role in liver carcinogenesis and chronic liver diseases, especially NASH and liver cirrhosis (8, 35).

The most clinically significant finding was the increased levels of AGEs as an independent factor for the prediction of DM development in these patients.

As mentioned above, AGEs activate multiple intracellular signaling pathways controlling different cellular functions, which in turn lead to pathophysiological effects (36–38). This is achieved through binding with RAGE triggering different signaling events such as protein kinase C (PKC) activation, nuclear factor kappa-B (NF-κB) accumulation and activation, reactive oxygen species (ROS) generation, and initiation of inflammatory signaling cascades such as the mitogen-activated protein kinase (MAPK) pathway, interleukin 6 (IL-6), tumor necrosis factor-alpha (TNF-α), and expression of intercellular adhesion molecule 1 (ICAM-1) and vascular cell adhesion molecule 1 (VCAM-1), which together result in the pathogenesis of several diseases (39). The interaction of AGEs with their specific RAGE has been proposed to play a critical role in several chronic diseases.

AGEs contribute to both IR and β-cell damage and death leading to impaired insulin function. Many studies investigated the cytotoxic potential of AGEs on pancreatic β-cells. Shu et al. (40) reported that Tribbles homolog 3 (TRB3) damages insulinoma cells (INS-1 cells), resulting in their apoptosis. TRB3 regulates nicotinamide adenine dinucleotide phosphate oxidase activity and activates the protein kinase C β2 pathway, inducing ROS synthesis and resulting in oxidative stress in INS-1 cells. In contrast, diminished cellular proliferation, by ROS-induced cell death in HIT-T15 cells due to treatment with ribose-modified serum, was observed by Puddu et al. (41). Moreover, Zhu et al. showed that RAGE may be the main cause of β-cell apoptosis characterized by caspase activation, cytochrome c release, and reduced anti-apoptotic bcl2 expression (42). Decreased insulin synthesis and reduced secretion are both involved in β-cell failure contributing to hyperglycemia. The most interesting aspect of our data is the inverse correlation between AGE levels and HOMA-β. According to this, we can infer that AGEs play a major role in β-cell dysfunction.

Hepatitis C viral protein favors IR, which plays a crucial role in accelerating hepatic fibrosis and increasing its severity in infected patients (14). However, in non-diabetic patients, the AGE serum level is linked to both HOMA-IR and liver stiffness (29). This also accords with our observations, which showed that the AGE level is correlated with HOMA-IR as well as CTP and MELD classifications.

AGEs alter insulin signaling through many mechanisms. They modify the insulin directly, which in turn alters its action resulting in impaired glucose uptake, reduced insulin clearance, or further increased insulin secretion. They increase RAGE expression and decrease NAD-dependent deacetylase sirtuin-1 (SIRT1) expression, which alters its signaling and induces inflammation. They stimulate PKC β and upregulate TNF-α (43, 44).

If HCV caused the increase in AGE levels (29), they should have returned to their normal levels after treatment with direct-acting antivirals (DAAs). However, we did not observe any decrease in the measurements of their levels after therapy and follow-up in comparison to the baseline levels for these patients. This means that liver cirrhosis is the only factor behind this elevation.

Contrary to expectations, this study did not find a significant correlation between HCV therapy with DAAs and DM development in patients with cirrhosis, while in non-diabetic lean patients, therapy of HCV enhances peripheral (but not hepatic) insulin sensitivity in CHC without significant fibrosis (45).

The principal theoretical implication of this study is that several other aspects besides HCV infection must be kept in mind when interpreting variables of glucose homeostasis in such patients, e.g., AGEs, IR, and the improvement in perceived well-being throughout therapy, which led to the adoption of a more dynamic lifestyle by these patients (46).

The AGE–RAGE axis is an important player in HSC activation and resulting liver fibrosis. Reducing the AGE–RAGE signaling, by controlling high glucose, sensitizing insulin function, avoiding overcooked foods, and oxidant supplement digestion (47), is a promising method in lowering the risk of DM and liver fibrosis in CHC patients, especially with increased AGEs and IR.

The most striking result to emerge from the data is that age may be considered as an independent factor for the prediction of DM development in this study. This is probably because insulin sensitivity decreases with age and compensation of β-cell function becomes insufficient in the face of increasing IR (48). This state of reduced β-cell proliferation capacity and higher sensitivity to apoptosis is related to aging (49). Oxidative stress, decreased neuronal stimulation, subclinical inflammation, and IR are the main pathophysiological causes of sarcopenia (generalized and progressive muscle mass loss with age). These conditions contribute to the development of glucose intolerance and type 2 diabetes (50). These results reflect those of Mehta et al. who also stated that 40 year-old people with CHC are more prone to develop DM (51).

This study, as far as we know, is the first to add to this expanding research field by uncovering the impact of AGEs in such patients. These findings have significant implications in the understanding of the involvement of AGEs in the development of DM.

There are certain limitations to generalize these results: first, small sample size; second, single-center study; third, the cause of liver cirrhosis is solely HCV; fourth, the effect of liver transplantation on the levels of AGEs and the development of DM in such patients (52); fifth, the follow-up period was only 2 years. Finally, these results need to be confirmed using formal methods for assessing insulin secretion and IR, such as the intravenous glucose tolerance test and the hyperinsulinemic clamp.

Despite these limitations, HOMA-β and HOMA-IR are the most broadly utilized markers for β-cell function and IR in epidemiological studies and are widely used for comparing insulin secretion and IR among various population-based studies (53).

In conclusion, AGEs were evidently related to increased incidence of DM, especially in patients with liver cirrhosis. This may be a simple yet effective method to lower the risk of DM and liver fibrosis in these patients.

## Data Availability Statement

The data that support the findings of this study have restrictions and so are not publicly available. Data are however available from the authors upon reasonable request.

## Ethics Statement

The studies involving human participants were reviewed and approved by Mansoura Institutional Review Board. The patients/participants provided their written informed consent to participate in this study.

## Author Contributions

AA-R, NM, and SZ contributed to the concept and/or design of the study and performed the statistical analysis. MosA, ME, and MohA contributed to the acquisition of the data, interpreted data critically, and revised the manuscript. AH drafted the manuscript. RasE critically revised the manuscript and recruited and followed up with patients. RanE acquired, analyzed and interpreted data, and revised the manuscript. WE and NE-W contributed to the analysis and interpretation of data and performed the statistical analysis. All authors approved the final version of the article, including the authorship list.

## Conflict of Interest

The authors declare that the research was conducted in the absence of any commercial or financial relationships that could be construed as a potential conflict of interest.

## Abbreviations

AGEs: advanced glycation end products
DM: diabetes mellitus
HD: hepatogenous diabetes
BMI: Basal metabolic index
RAGE: Receptor for Advanced Glycation Endproducts
eGFR: estimated glomerular filtration rate
HOMA-IR: Homeostatic Model Assessment-Insulin Resistance
HCV: hepatitis C virus
SOF: Sofosbuvir
DCV: daclatasvir
IFG: impaired fasting glucose
CTP: Child-Turcotte Pugh
MELD: Model for End-Stage Liver Disease
NASH: nonalcoholic steatohepatitis
CBC: complete blood count
TG: triglycerides
GPAQ: Global Physical Activity Questionnaire
PKC: protein kinase C
NF-κB: nuclear factor kappa-B
ROS: reactive oxygen species.
